# Pathogenicity of exopolysaccharide-producing *Actinomyces oris* isolated from an apical abscess lesion

**DOI:** 10.1111/j.1365-2591.2012.02099.x

**Published:** 2012-08-17

**Authors:** K Yamane, T Nambu, T Yamanaka, K Ishihara, T Tatami, C Mashimo, C B Walker, K-P Leung, H Fukushima

**Affiliations:** 1 Department of Bacteriology, Osaka Dental UniversityOsaka, Japan; 2 Department of Oral Biology, College of Dentistry, University of FloridaGainesville, FL, USA; 3 US Army Dental and Trauma Research Detachment, Institute of Surgical ResearchFort Sam Houston, TX, USA

**Keywords:** *Actinomyces oris*, apical abscess, biofilm, exopolysaccharide

## Abstract

**Aim:**

To demonstrate a capacity for producing exopolysaccharides (EPSs) and an ability to form biofilm on abiotic materials of *Actinomyces oris* strain K20.

**Methodology:**

The productivity of EPSs and the ability to form biofilm of strain K20 were evaluated by measuring viscosity of spent culture media and by scanning electron microscopy (SEM) and the biofilm assay on microtitre plates, respectively. High-performance liquid chromatography was used to determine the chemical composition of the viscous materials. To examine the role of the viscous materials attributable to the pathogenicity in this organism, the ability of strain K20 to induce abscess formation was compared in mice to that of ATCC 27044.

**Results:**

The viscosity of the spent culture media of K20 was significantly higher than that of ATCC 27044. Strain K20 showed dense meshwork structures around the cells and formed biofilms on microtitre plates, whereas ATCC 27044 did not. Chemical analysis of the viscous materials revealed that they were mainly composed of neutral sugars with mannose constituting 77.5% of the polysaccharides. Strain K20 induced persistent abscesses in mice lasting at least 5 days at a concentration of 10^8^ cells mL^−1^, whereas abscesses induced by ATCC 27044 healed and disappeared or decreased in size at day 5.

**Conclusions:**

Strain K20 produced EPSs, mainly consisting of mannose, and formed biofilms. This phenotype might play an important role for *A. oris* to express virulence through the progression of apical periodontitis.

## Introduction

Bacteria remaining in the apical part of the root canal are known to be involved in the development of apical abscesses (Nair 2006, Siqueira & Rôças 2008); however, it is still unclear how these bacteria survive repeat root canal treatments. The ability to produce exopolysaccharides (EPSs) could contribute to their survival and the development of persistent infections in the human body (Costerton *et al*. 1999). For example, alginate is one of several biofilm matrices produced by *Pseudomonas aeruginosa*, a prototype biofilm-forming bacterium, and is responsible for the persistence (Ryder *et al*. 2007) and antibiotic tolerance of this organism in the airway of patients with cystic fibrosis (Shankar *et al*. 1995). Accumulated evidence emphasizes that EPSs production of oral bacteria is associated with persistent infections in the oral cavity (Yamane *et al*. 2009, 2010, Yamanaka *et al*. 2010). *Capnocytophaga ochracea* found in periodontal disease has been shown to produce mannose-rich EPS that can suppress murine lymphocyte mitogen responses and activate human complement response (Bolton & Dyer 1983, 1986, Dyer & Bolton 1985). It was reported that some clinical isolates of *Prevotella intermedia* and *P. nigrescens* isolated from chronic periodontitis lesions produce EPSs in a sucrose-independent manner and form biofilms that contribute to their virulence (Fukushima *et al*. 1992, Yamane *et al*. 2005, Yamanaka *et al*. 2009). Clinical strains of *Bacillus subtilis*, *Rothia mucilaginosa* and *Escherichia hermannii* isolated from persistent periapical lesions have an ability to produce self-synthesized EPSs, suggesting that these organisms can cause a persistent biofilm infection in the apical region of root canal (Yamane *et al*. 2009, 2010, Yamanaka *et al*. 2010). Previous studies using scanning electron microscopy (SEM) revealed that a significant number of microorganisms were embedded in the extracellular polymeric substances in apical periodontitis lesions (Fukushima *et al*. 1990, Nair 2006, Rocha *et al*. 2008). These studies also suggested that EPSs-producing bacteria in the periapical lesion play an important role in the recurrence of acute apical periodontitis or in the development of abscess lesions from asymptomatic apical pathosis.

In this study, the unique characteristics of *Actinomyces oris* strain K20 that was isolated as a dominant bacterium in an apical abscess lesion and has been maintained in a culture collection since its initial isolation have been described. This strain spontaneously formed mucoid-type colonies despite subculturing under laboratory condition and produces viscous materials in spent culture medium. Here, the physical characteristic (viscosity) and chemical nature of the EPS produced by this organism, its ability to form biofilm and its pathogenicity as determined by abscess formation in mice, were characterized. Additionally, re-identification was performed on this clinical strain because it has been reported that strains of *A. naeslundii*, *A. viscosus*, *A. johnsonii* and *A. oris* cannot be identified by conventional phenotype testing and 16S ribosomal ribonucleic acid (rRNA) sequencing (Henssge *et al*. 2009).

## Materials and methods

### Bacteria

A clinically isolated *A. oris* strain K20 from an apical abscess lesion was used. Strain K20 was found to be one of the dominant bacteria in an oral abscess lesion and identified as *A. oris* by phylogenetic analysis. The isolation of a bacterium from an apical abscess was performed according to routine procedures as follows: The patient was asked to rinse the mouth with 0.2% chlorhexidine solution for 30 s. The apical abscess (submucosa abscess) was then isolated with sterile cotton wool rolls, and the overlying mucosa was wiped with a cotton wool bud soaked in 0.2% chlorhexidine solution. The suppuration sample was collected from the apical abscess by aspiration with a sterile 21-gauge needle. The sample was diluted with 0.1 mol L^−1^ phosphate-buffered saline (PBS) and plated on anaerobe 5% sheep blood agar plates (BAPs) formulated by the Center for Disease Control (BBL Microbiology Systems, Cockeysville, MD, USA). The inoculated plates were incubated anaerobically with the atmosphere composed of 10% carbon dioxide (CO_2_), 10% hydrogen (H_2_) and 80% nitrogen (N_2_) in an anaerobic chamber (ANX-3, Hirasawa, Tokyo, Japan) or aerobically at 37 °C for 24–72 h. Grown colonies were used to recover biofilm-forming bacteria. The samplings of bacteria were from patients who were referred to the Department of Endodontics at Osaka Dental University Hospital and approved by the ethics committee of Osaka Dental University (Ethics Committee Approval No. 060641). Informed consent was obtained from the patients after the nature of the procedure, possible discomfort and risks had been fully explained.

### Re-identification of strain K20 by phylogenetic analysis using *atpA* sequence

Strain K20 was presumptively identified as *A. viscosus* by 16S rRNA gene sequencing and catalase productivity (Ellen 1976). However, a recent study suggested that partial sequences of two housekeeping genes (*atpA* and *metG*) can separate *A. naeslundii* genospecies 1, genospecies 2 (including *A. viscosus* serotype 2) and genospecies 3 (Henssge *et al*. 2009). Therefore, pyrosequencing of strain K20 genome was performed to obtain data necessary for re-identification of this organism. A draft genome sequence of strain K20 was determined with a combined strategy of 454-pyrosequencing and paired-end sequencing using the Roche Genome Sequencer FLX System (Roche Diagnostics, Tokyo, Japan) and Applied Biosystem 3730 DNA (deoxyribonucleic acid) Analyzer (Applied Biosystems, Tokyo, Japan). The sequence data were assembled with GS *De Novo* Assembler Software (version 1.1.03.24) (Roche Diagnostics) and deposited in the databases of the DNA Data Bank of Japan (DDBJ) (accession number: BABV01000001-BABV01000771, http://www.ddbj.nig.ac.jp/index-e.html).

**Figure 1:**
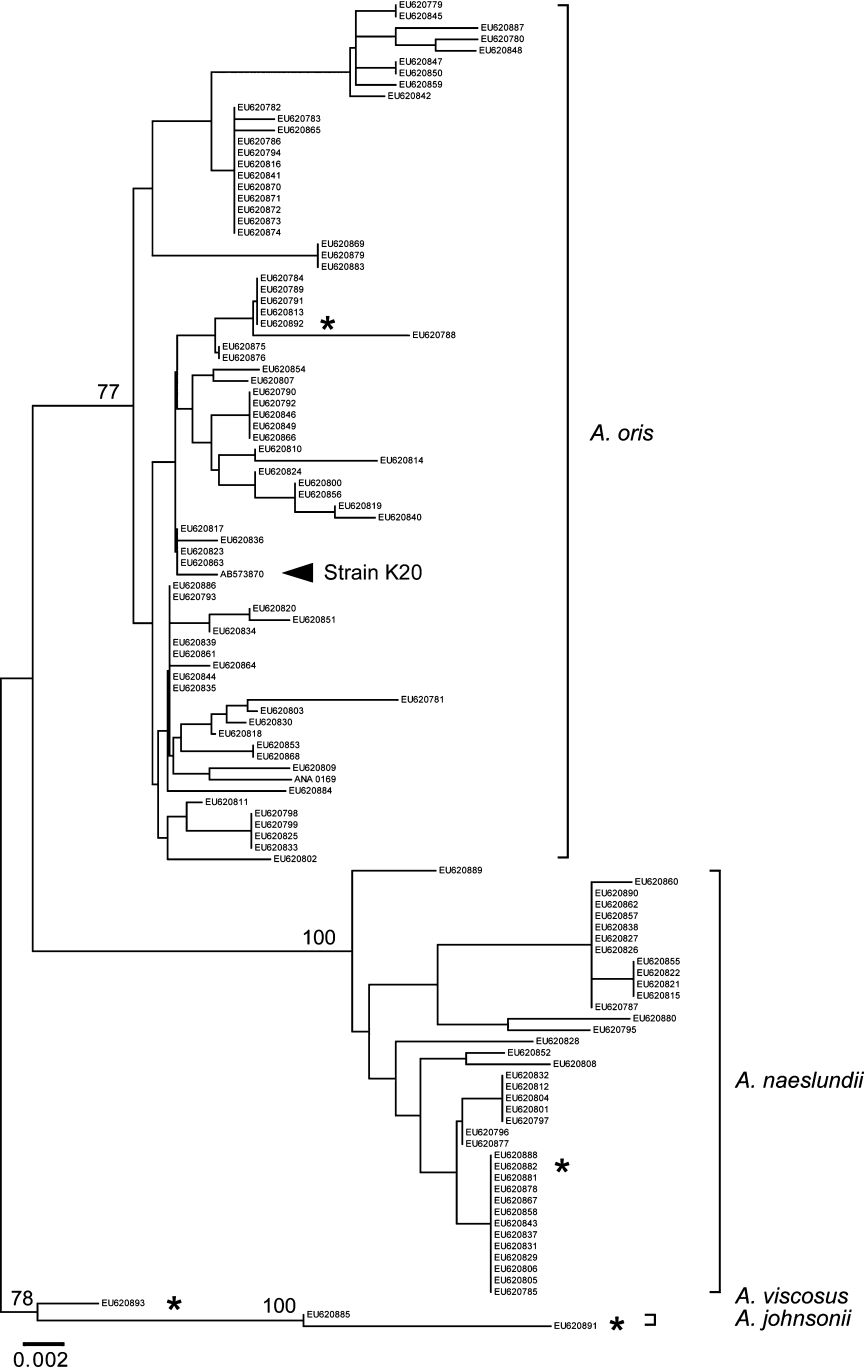
Phylogenetic tree based on *atpA* region for *Actinomyces* species. A data set provided by Henssge *et al*. (2009) was used (accession numbers are indicated at the tree). Four clusters, each cluster containing a representative type strain (asterisk), were marked. The scale bar represents a difference of 0.002 substitution per site.

Phylogenetic analysis was performed by using the sequence of the housekeeping gene *atpA* (ATP synthase F1 alpha subunit: accession number: AB573870, DDBJ) and the data set of *atpA* for *Actinomyces* as described elsewhere (Henssge *et al*. 2009). The nucleotide sequences of *atpA* were aligned by MAFFT (version 6) with FFT-NS-I option (Katoh & Toh 2010). Then, a neighbour-joining tree was constructed using Jukes–Cantor distance matrices. Bootstrap values were determined with 500 replicates. A phylogenetic tree constructed in this study indicated that strain K20 belonged to the *A. oris* clade with high bootstrap values (Fig. 1). Therefore, *A. oris* ATCC 27044 (an ATCC type strain for *A. oris*) was chosen as a reference strain for comparison in this study.

### Viscosity of spent culture media

The viscous material- or EPS-producing ability of strains K20 and ATCC 27044 was first evaluated by measuring the viscosity of spent culture media. Strains K20 and ATCC 27044 were grown in tryptic soy broth (TSB) for 20 h. For the viscosity measurement, an automated microviscometer (AMVn) (Anton Paar, Graz, Austria) was used in accordance with the manual provided by the manufacturer. The culture supernatant was filled into a glass capillary and maintained at 20 °C. The measurement was performed five times on each sample, and the results of four independent cultures were averaged to give the final value.

### SEM observation

SEM was used to observe the cell surface structure of strains K20 and ATCC 27044. Each strain grown on a BAP aerobically for 24 h was collected on a piece of filter paper (Glass fiber GA55, Toyo Roshi, Tokyo, Japan) and fixed with 2% glutaraldehyde in 0.1 mol L^−1^ PBS (Nissui Pharmaceutical, Tokyo, Japan) for 2 h followed by 1% osmium tetroxide (OsO_4_) in 0.1 mol L^−1^ PBS for 1 h at 4 °C. Samples were dehydrated and followed by platinum ion coating (E-1030, Hitachi, Tokyo, Japan). Specimens were examined with an SEM (S-4800, Hitachi) at an accelerating voltage of 1 kV.

### Biofilm assay

The ability to form biofilm was determined as described previously (O'Toole *et al*. 2000). Briefly, strains K20 and ATCC 27044 were inoculated into 1 mL of *Actinomyces* broth (BBL Microbiology Systems) in 24-well flat-bottomed microplates (AGC Techno Glass, Chiba, Japan) at a concentration of 10^7^ CFU per well. The plates were incubated statically for 2 days at 37 °C without shaking to establish biofilms. Unbound cells were removed by inversion of the microplate and tapping on an absorbent paper towel. Adherent cells were then stained with 0.1% crystal violet for 15 min. Excess stain was removed by washing with PBS. The stained wells were recorded photographically using a camera set (PC1089, Canon, Tokyo, Japan). The crystal violet was then solubilized by the addition of 95% ethanol, and absorbance at 570 nm was measured by spectrophotometer U-2000 (Hitachi). Quadricate wells for each strain were measured, and the experiments were performed in triplicate.

### Preparation of EPSs

The EPS (viscous materials) was prepared from culture supernatants by the method described by Campbell & Pappenheimer (1966). Briefly, strains K20 and ATCC 27044 were grown at 37 °C in TSB for 24 h, respectively. Supernatants were separated by centrifuging the liquid culture at 8,000 rpm for 30 min, and sodium acetate was added to supernatants at a final concentration of 5%. The mixture was stirred for 30 min at 22 °C, and the EPS was isolated by ethanol precipitation from the reaction mixture. The ethanol-precipitated material was collected by centrifugation (12,000 rpm for 15 min, at 22 °C), resolved in 5% sodium acetate and then treated with chloroform–1-butanol (1:5, by volume). Water-soluble and chloroform–butanol layers were separated by centrifugation, equal amounts of ethanol were added to the water-soluble layer, and the ethanol-precipitated material was freeze-dried until use.

### High-performance liquid chromatography (HPLC) analysis of EPSs

Neutral monosaccharides were released from purified EPS (5 mg) by hydrolysis in a sealed tube with 2 N trifluoroacetic acid (200 mL) at 100 °C for 6 h. The hydrolysate was concentrated *in vacuo* and dissolved in 500 mL of distilled water. The sugars were identified by HPLC (LC-9A, Shimadzu, Kyoto, Japan) with a TSKgel® sugar AXG column (15 cm × 4.6 mm) (Tosoh, Tokyo, Japan) using 0.5 mol L^−1^ potassium tetraborate buffer (pH 8.7) as a carrier at a flow rate of 0.4 mL min^−1^ and a column temperature of 70 °C. The amount of extracellular DNA in purified viscous materials or EPSs (1 mg) was determined by absorbance measurement at 190–360 nm with a U-2001 spectrophotometer (Hitachi).

### Animal study

Strains K20 and ATCC 27044 were grown in TSB for 48 h. The optical density of each bacterial culture was measured at 600 nm, and their cell concentrations were adjusted to 5.0 × 10^8^ CFU mL^−1^ according to a standard curve. A total of 500 μL of bacteria was injected subcutaneously into the inguinal region of each BALB/c mouse (male, 4 weeks, five mice per strain). The development of abscess lesions was recorded photographically using a camera (Nikon FIII, Nikon, Tokyo, Japan) set at a fixed magnification for five consecutive days. Sizes of abscess areas were determined with Photoshop (version 7.0, Adobe, San Jose, USA) and expressed in number of pixels. Statistical analysis was performed with Student's *t*-test. This examination was repeated three times. The animal protocol used in this study was approved by the Animal Research Committee of Osaka Dental University (Confirmation numbers 05-21001 and 06-05003).

## Results

### Re-identification of strain K20

The phylogenetic tree constructed with the data set of *atpA* for *Actinomyces* spp. (Henssge *et al*. 2009) and the sequence of *atpA* of strain K20 obtained by the genome pyrosequencing of strain K20 indicated that it belonged to the *A. oris* clade as described previously (Fig. 1).

### Viscosity of spent culture medium

**Figure 2:**
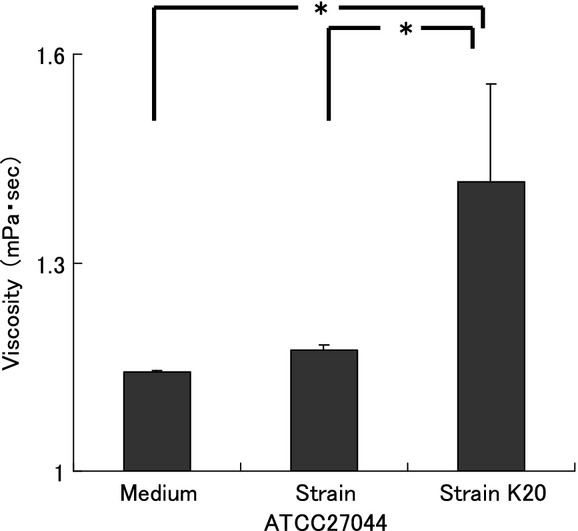
Viscosities of spent culture media obtained from strains K20 (a clinical isolate from an apical abscess lesion) and ATCC 27044 (a reference strain for *Actinomyces oris*). Medium: control tryptic soy broth without bacterial inoculation. Statistical analysis was performed with Student's *t*-test. *P < 0.05.

The viscosity of the spent culture medium obtained from strain K20 cultures was significantly higher than that of the control TSB medium or ATCC 27044 cultures (Fig. 2).

### Chemical compositions of viscous materials (EPSs)

Chemical analyses showed that the purified viscous materials obtained from strain K20 were composed of neutral sugars with mannose constituting 77.5% of sugars recovered from the culture supernatant (Table 1), suggesting that these materials are EPSs. This fraction did not contain extracellular DNA at a substantial level (data not shown). Viscous materials were not isolated from the spent culture of ATCC 27044 at a substantial level.

**Table 1:** Sugar composition of exopolysaccharides isolated from strain K20

Neutral sugars	Contents (μg)	Content ratio (%)
Mannose	165.31	77.5
Glucose	21.71	10.2
Ribose	13.28	6.2
Galactose	6.15	2.9
Fructose	3.32	1.6
Xylose	2.10	1.0
Arabinose	1.14	0.5
Rhamnose	<1.00	<0.5
Total	213.28	100

### SEM observation of cell surface structures

**Figure 3:**
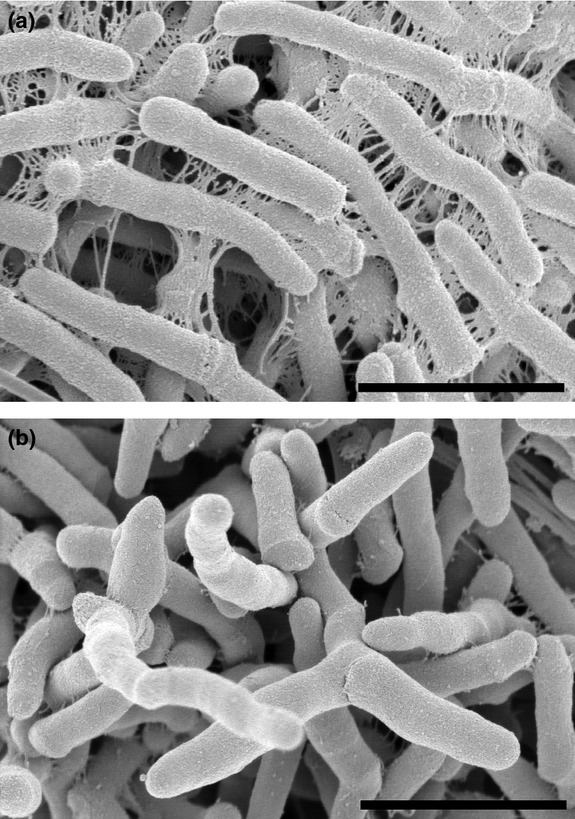
Scanning electron micrographs showing the surface structure of *Actinomyces oris* strains K20 (a) and ATCC 27044 (b). Meshwork-like structures were found on the cell surfaces of strain K20 (a), but not on strain ATCC 27044 (b). Bars = 2.0 μm.

SEM revealed that strain K20, but not ATCC 27044, had dense meshwork-like structures around the cells (Fig. 3).

### Biofilm formation in microtitre plates

**Figure 4:**
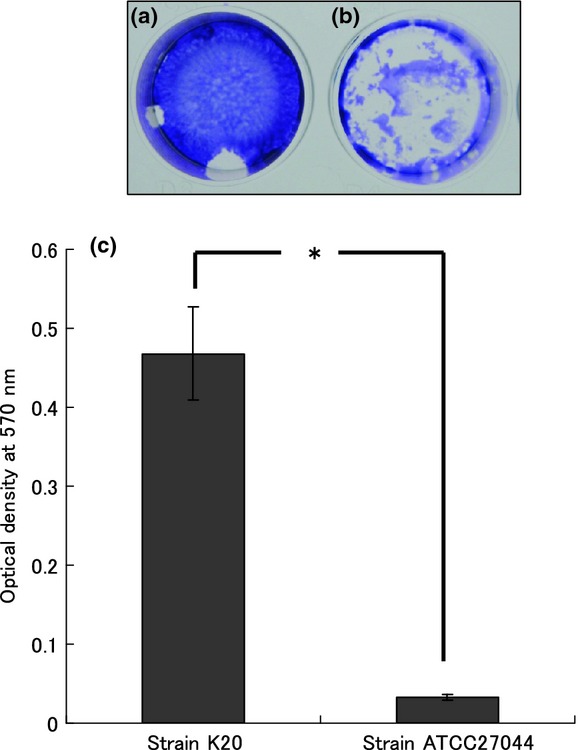
Biofilm formations on microtitre plates. Crystal violet was used to stain biofilm materials. Strain K20 formed thick biofilm in the well (a), but ATCC 27044 did not (b). The quantitative analysis of biofilm production measured the optical density of destained biofilms at 570 nm (c). Statistical analysis was performed with Student's *t*-test. Bars indicate standard deviation. *P < 0.01. The data represent one of three independent experiments.

The ability to form biofilm was investigated for strains K20 and ATCC 27044 using a crystal violet microtitre plate assay. Figures 4 (a,b) show that strain K20 was able to form biofilm on polystyrene plates, whereas ATCC 27044 showed poor biofilm formation. Quantitative analysis measuring the optical density of destained biofilms at 570 nm revealed that the ability of strain K20 to form biofilm was significantly greater than that of ATCC 27044 (Fig. 4c).

### Animal study

**Figure 5:**
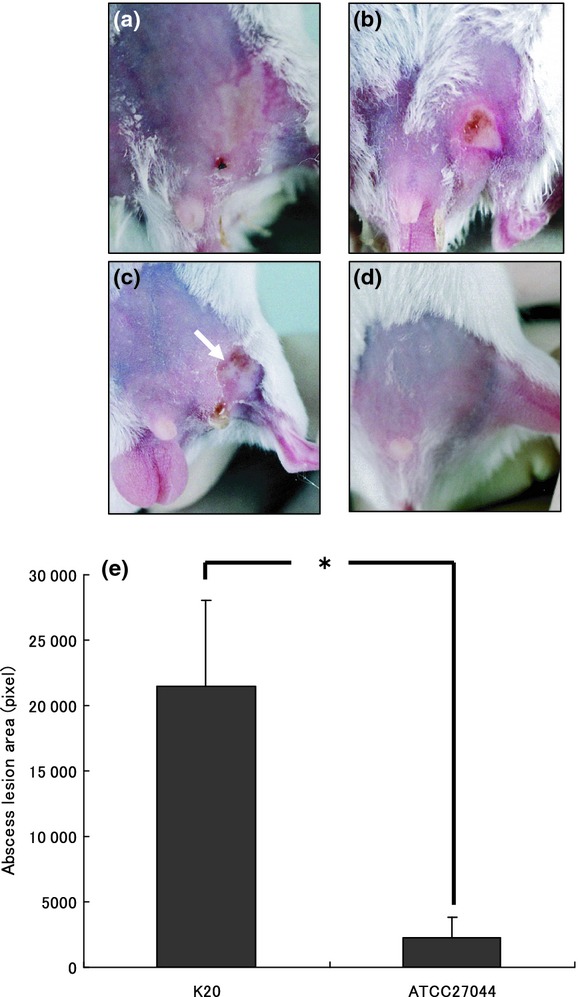
Abscess induction in mice by strains K20 and ATCC 27044. Two days after the injection of suspended bacterial cells at a concentration of 10^8^ CFU mL^−1^, strains K20 and ATCC 27044 induced distinct abscesses in mice (a,b). Persistent abscesses (white arrows) were observed at day 5 (c). In contrast, the abscess lesions induced by ATCC 27044 decreased in size or cured and disappeared (d). The average abscess size induced by strain K20 was significantly larger than that of ATCC 27044 (e). Statistical analysis was performed with Student's *t*-test. *P < 0.05.

Two days after the injection of suspended bacterial cells at a concentration of 10^8^ CFU mL^−1^, strains K20 and ATCC 27044 induced distinct abscesses in mice (Fig. 5a,b). The abscesses induced by strain K20 lasted at least 5 days in all mice, whereas the abscess lesions induced by ATCC 27044 healed and disappeared in three of five mice or decreased in size in the other two mice at day 5 (Fig. 5c,d). The average abscess size induced by strain K20 at day 5 was significantly larger than that of ATCC 27044 (Fig. 5e).

## Discussion

*Actinomyces* spp. are predominant members of human oral commensal microbiota and are known as initial colonizers on tooth surfaces (Nyvad & Kilian 1987, Kolenbrander *et al*. 2005, Diaz *et al*. 2006). Oral *Actinomyces* spp. are branching, non-spore-forming rods that belong to the high G + C division of Gram-positive bacteria. On the basis of 16S rRNA gene sequencing and DNA–DNA relatedness, six oral *Actinomyces* spp. (*A. georgiae, A. odontolyticus, A. israelii, A. gerencseriae, A. meyeri and A. naeslundii* genospecies I, II and III) have been recognized (Tanner *et al*. 1994). The identification of *A. naeslundii* strains, including *A. viscosus* serotype 2 strains, had been problematic because neither 16S rRNA gene sequencing nor biochemical tests can differentiate these three genospecies. Recently, Henssge *et al*. (2009) demonstrated that the partial sequences of two housekeeping genes (*atpA* and *metG*) can separate three *A. naeslundii* genospecies. They proposed the name *A. oris* for *A. naeslundii* genospecies II and the name *A. johnsonii* for *A. naeslundii* genospecies III (genospecies WVA 963). In this classification, *A. naeslundii* genospecies I remains as *A. naeslundii*. According to this classification, strain K20 was re-identified as *A. oris*.

In persistent apical periodontitis lesions, actinomycosis has been known as an important cause (Nair & Schroeder 1984), and several oral *Actinomyces* spp. such as *A. israelii* (Nair 2006), *A. radicidentis* (Collins *et al*. 2000, Kalfas *et al*. 2001) and *A. naeslundii* (Happonen *et al*. 1985) are frequently isolated from human apical periodontitis lesions. Figdor *et al*. (1992) demonstrated that the pathogenicity of *A. israelii* isolated from a case of failed endodontic therapy (Sundqvist & Reuterving 1980) was because of its ability to establish characteristic cohesive colonies consisting of cells enmeshed in an extracellular matrix. The organisms existing in such colonies can collectively evade destruction and elimination by host phagocytic cells. A recent study demonstrated the capacity of *A. israelii* to produce EPSs and to form biofilm *in vitro* on copper surfaces of intrauterine contraceptive devices immersed in a simulated uterine fluid under anaerobic conditions (Carrillo *et al*. 2010). *Actinomyces radicidentis* is known to form biofilms, in which bacteria seem to be embedded in the extracellular matrix and exhibit antiphagocytic activity against rat neutrophils (Nair *et al*. 2008). *Actinomyces meyeri*, the cause of various actinomycosis or abscess lesions (Apothéloz & Regamey 1996, Liaudet *et al*. 1996), was also found in periapical lesions refractory to endodontic therapy (Sunde *et al*. 2002).

The present study found that *A. oris* strain K20 has a capacity to produce EPSs in liquid culture condition as viscous materials and meshwork structures around the cells on agar plates. These activities are similar to those of slime-producing bacteria with biofilm-forming ability such as *P. aeruginosa* (Kobayashi 1995, Yasuda *et al*. 1999), *Salmonella enterica* Serovar Typhimurium (Anriany *et al*. 2001), *Staphylococcus epidermidis* (Vuong *et al*. 2004) and *Vibrio cholerae* (Wai *et al*. 1998). Considering the fact that ATCC 27044, a type strain for *A. oris*, which lacks an ability to produce EPS at a substantial level and does not form meshwork on its cell surface, failed to form apparent biofilm on microtitre plates and could not induce persistent abscess lesions in mice, the self-synthesized polysaccharide seems to be important to biofilm formation on abiotic materials as well as to the pathogenicity of *A. oris* strain K20. It is important to note that other constituents such as extracellular nucleic acids (Wu & Xi 2009) or secreted proteins (Latasa *et al*. 2006) found in other organisms are also used as part of the biofilm matrix that are essential in their biofilm development. Similar tubule-like structures are formed by bacterial nanotubes (Dubey & Ben-Yehuda 2011) or amyloids (Dueholm *et al*. 2010). Several *Actinomyces* spp. are armed with type 1 and type 2 fimbriae for being able to adhere to a host surface, and fimbriated strains including *A. naeslundii* genospecies 1 (*A. naeslundii*), genospecies 2 (*A. oris*) and *A. viscosus* exert higher cell surface interactive force than those devoid of fimbriae (Tang *et al*. 2004). Therefore, how the EPSs produced by *A. oris* interacts with other cell surface structures and how many structural elements contribute to the establishment of persistent infection caused by *A. oris* are still to be elucidated. A study in the early 1970s clearly showed that the addition of slime from *P. aeruginosa* cultures to *E. coli* or *Staphylococcus aureus* dramatically inhibited phagocytosis by neutrophils (Schwarzmann & Boring 1971). As described previously, it has also reported that a clinical isolate of *P. nigrescens*, which has the ability to produce a copious amount of EPS, is far more potent in causing abscess formation in mice and far more resistant to the phagocytic activity of human polymorphonuclear leucocytes than the chemically induced mutant that lacked the EPS (Yamane *et al*. 2005). When the purified EPS from the biofilm-forming strain of *P. nigrescens* was added to the cultures of a biofilm-non-forming mutant, these cells exhibit the presence of these meshwork structures on their surfaces and demonstrate the ability to induce abscess formation in mice (Yamane *et al*. 2005). As *A. israelii* packed in alginate gel induced actinomycotic lesions in mice efficiently, but a bacterial suspension without alginate or gel particles without bacteria did not induce lesions (Sumita *et al*. 1998), it seems that polysaccharide coating of bacterial mass might determine the persistence of *Actinomyces* spp. in the host as described in many pathogens (Costerton *et al*. 1987).

Although the bacteria in biofilms are considered to be dormant in general, it is conceivable that *Actinomyces* spp. have another strategy to survive when the nutrients are scarce such as in apical lesions. Paddick *et al*. (2005) showed that *A. oris* (*A. naesulundii* genospecies 2) together with *Streptococcus oralis*, *Streptococcus mitis* and *Streptococcus intermedius* form the bulk of the microbiota that are present in dentine samples beneath the restoration for 5 months. *Actinomyces oris* in the 5-month-old dentine samples was positive for sialidase, β-galactosidase and *N*-acetyl-β-glucosaminidase activities (Paddick *et al*. 2005). The complete function of sialidase of this organism is not fully understood. Recent studies suggested that this enzyme could provide nutrients to the cells by liberating sialic acid from glycans (Paddick *et al*. 2005, Do *et al*. 2008) and mediate the adhesion of the bacteria to host tissues by exposing the galactose residues of glycoprotein glycans that are present on mucosal surfaces (Ruhl *et al*. 2000, Do *et al*. 2008). The importance of *A. oris* type 2 fimbriae, which possess the lectin-like activity, in biofilm formation and co-aggregation has been vigorously studied (Yeung 1999, Mishra *et al*. 2010).

The factors controlling the establishment of bacterial biofilm in the periapical lesion that can lead to the recurrence of an acute abscess lesion in the site appear to be complex. Further thorough investigations are needed to elucidate the nature of biofilm matrices, extracellular enzymatic activities and the interaction between the host and *A. oris* in the apical abscess lesion.

## Conclusions

Taken together, the results in this study suggest that the EPS productivity of *A*. *oris* strain K20 affects its pathogenicity and that this organism could be involved in the development of apical abscess lesions.
